# P-323. Examining Preliminary Adherence to Long-Acting Injectable Pre-Exposure Prophylaxis (LAI-PrEP) among Racial, Sexual, and Gender Minority Populations at NYC Health+Hospitals/Bellevue: The EquiPrEP Study

**DOI:** 10.1093/ofid/ofaf695.542

**Published:** 2026-01-11

**Authors:** Ofole Mgbako, Brandi Moore, Farzana Kapadia, Emma Kaplan-Lewis, Eunice Casey, Maria Khan, Sahnah Lim, Jason Felder, Katharine Ramos, Judith Ratcliffe, Robert Pitts

**Affiliations:** NYC Health+Hospitals, Brooklyn, NY; NYU School of Global Public Health, New York, New York; NYU School of Global Public Health, New York, New York; NYC Health and Hospitals, NY, New York; NYC Health and Hospitals, NY, New York; NYU Grossman School of Medicine, New York, New York; NYU Grossman School of Medicine, New York, New York; NYU Grossman School of Medicine, New York, New York; NYC Health+Hospitals, Brooklyn, NY; NYC Health+Hospitals, Brooklyn, NY; NYU Langone Health, New York, New York

## Abstract

**Background:**

The EquiPrEP study examines the effectiveness of equitable LAI-PrEP implementation in collaboration with community-based organizations (CBO) for populations with low PrEP use and high risk of HIV incidence, including Black/Latine cisgender men who have sex with men (BLMSM), Black/Latine cisgender women (BLCGW), and transgender/nonbinary persons (TGNB). Here, we present 6-month preliminary adherence outcomes and reasons for LAI-PrEP discontinuation.

**Table 1:**
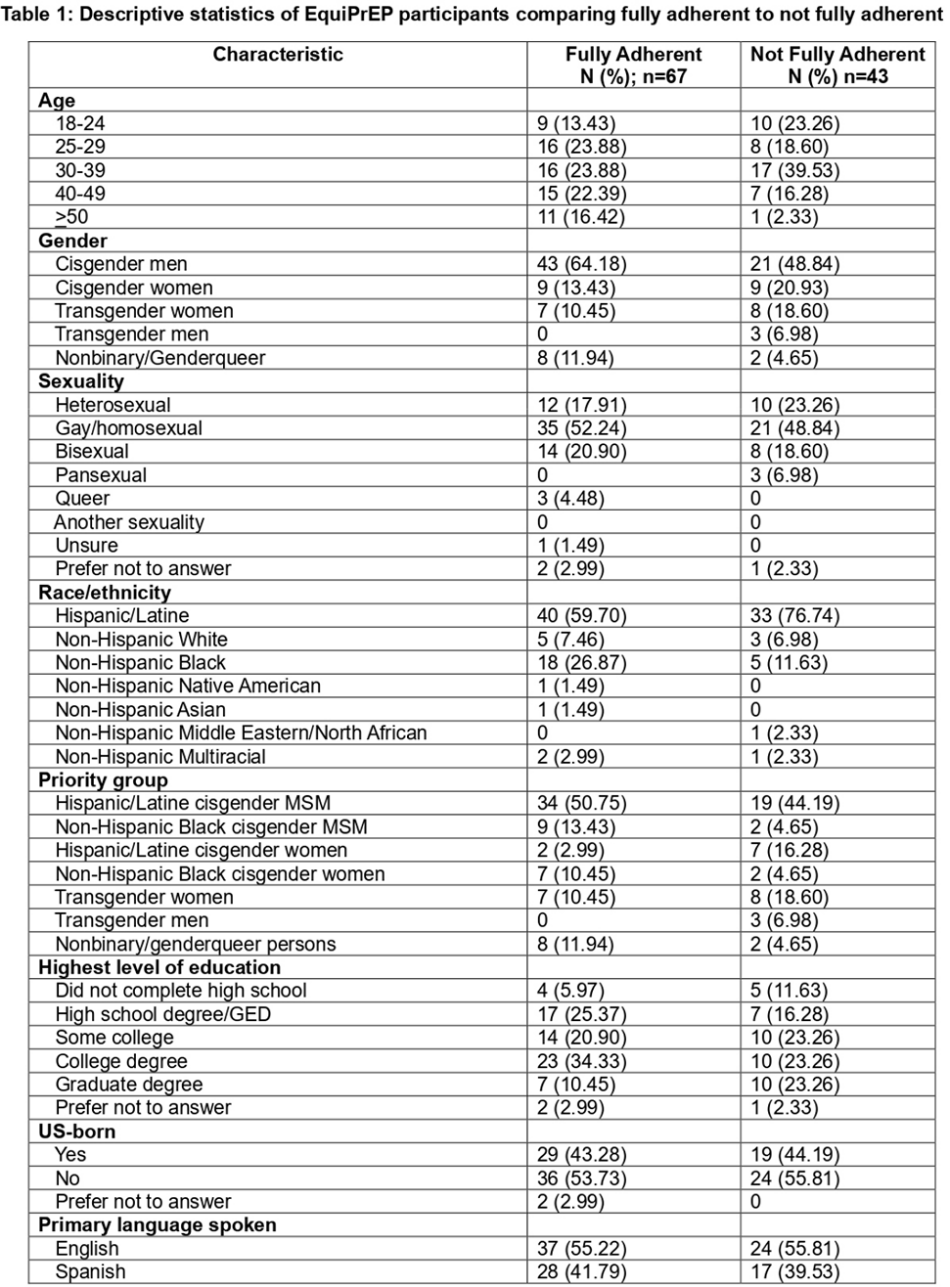
Descriptive statistics of EquiPrEP participants comparing fully adherent to not fully adherent

**Table 1:**
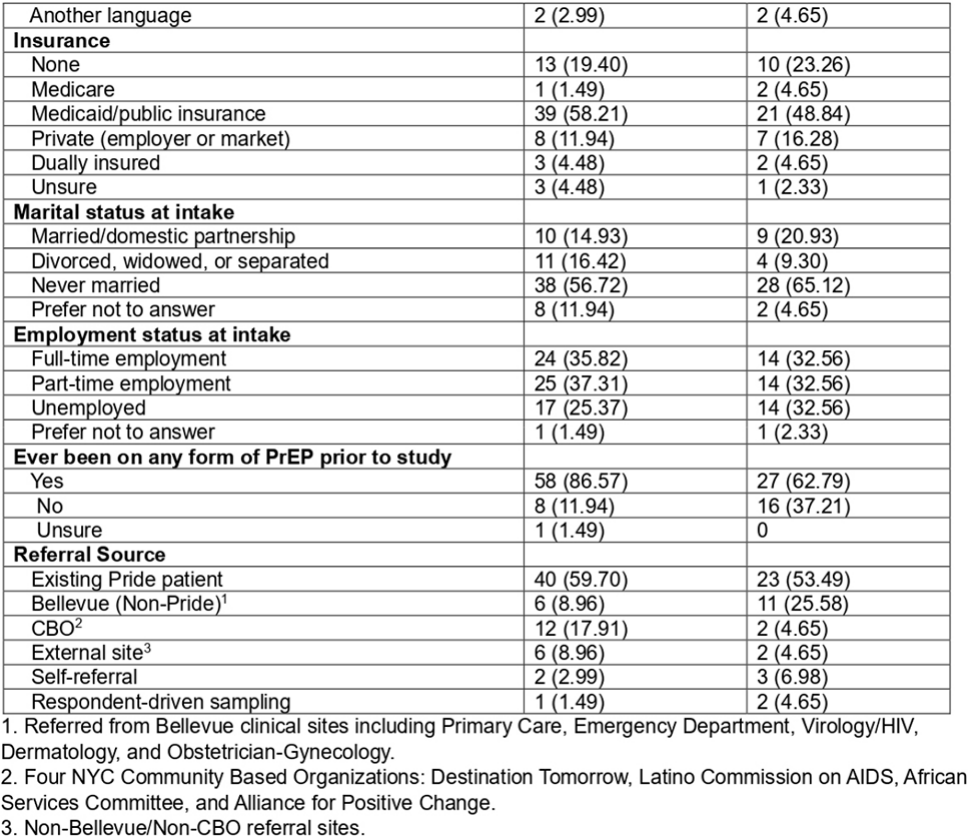
(Continued): Descriptive statistics of EquiPrEP participants comparing fully adherent to not fully adherent

**Methods:**

Data were collected between 2/2023-12/2024 at Bellevue Hospital Pride clinic within NYC Health+Hospitals, the largest public hospital system in the US. Patients included were:18yo, HIV-seronegative, and identified as BLMSM, BLCGW, or TGNB. Baseline and follow-up surveys collected information on socio-demographics, prior PrEP status, referral source and reason for LAI-PrEP discontinuation. Full adherence was defined as all injections occurring on-time within 6 month follow up. On-time injections were defined as the initial 2 doses occurring 4 weeks apart and follow up injections every 8 weeks (+/- 7 days). Descriptive statistics were used to characterize LAI-PrEP adherence and nonadherence.

**Table 2:**
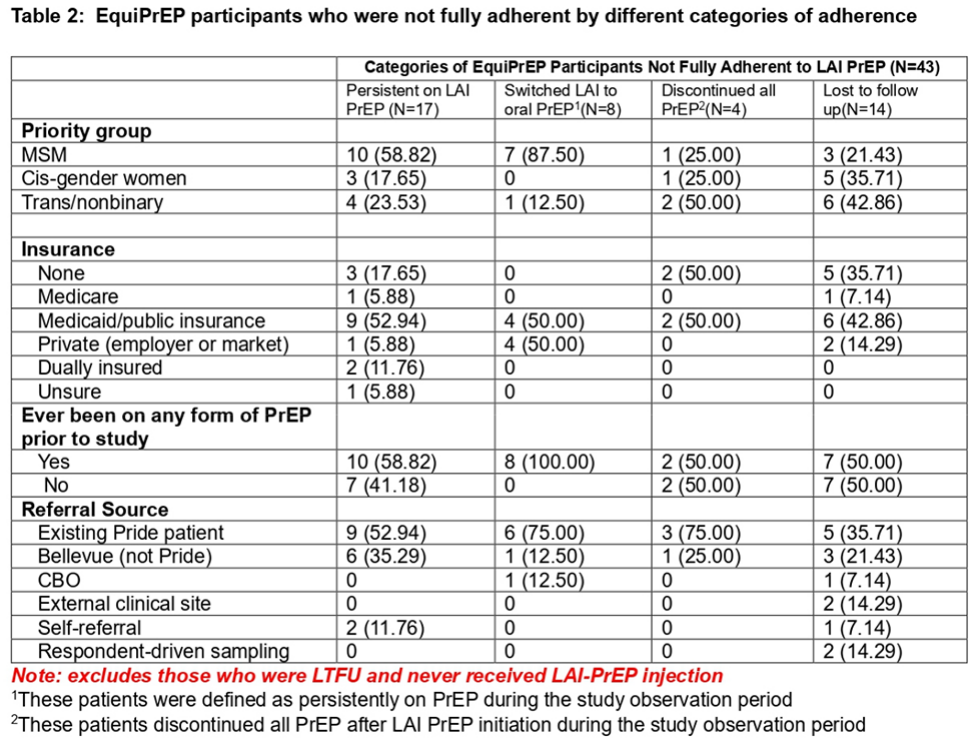
EquiPrEP participants who were not fully adherent by different categories of adherence

**Results:**

Of 110 participants enrolled who had 6-months of follow-up data, 60.9% (n=67) were fully adherent and 39.1% (n=43) were not fully adherent. Participants who were BLMSM, Black CGW or NB, on Medicaid, employed full/part-time, using any prior form of PrEP, or an existing patient at Bellevue/referred from a community based organization were more likely to be fully adherent (Table 1). Of those not fully adherent, 15.5% (n=17) continued on LAI-PrEP over 6 months, 7.3% (n=8) switched to oral PrEP, 3.6% (n=4) discontinued all PrEP, and 12.7% (n=14) were lost to follow up.

**Conclusion:**

Over 83% of participants were either fully adherent, continued on LAI-PrEP, or switched to oral PrEP over 6 months, demonstrating the potential for successful implementation of overall PrEP coverage for priority populations to reduce HIV vulnerability in partnership with CBOs. Participants discontinuing all PrEP or lost to follow up likely need enhanced support to decrease structural barriers to adherence. Future studies should focus on long-term sustainability of LAI-PrEP adherence.

**Disclosures:**

Ofole Mgbako, MD, MS, Gilead Sciences: Advisor/Consultant Emma Kaplan-Lewis, MD, gilead: Grant/Research Support Robert Pitts, MD MPH, Gilead Inc: Advisor/Consultant|ViiV: Advisor/Consultant

